# Dietary Taurine Supplementation Improves Sperm Quality and Modulates Seminal Plasma Metabolism in Heat-Stressed Dairy Goat Bucks

**DOI:** 10.3390/ani16071086

**Published:** 2026-04-01

**Authors:** Tingshu Fu, Mengwei Chen, Ying Pan, Xueqin Wang, Xiaonan Bai, Menghao Pan, Baohua Ma, Sha Peng

**Affiliations:** College of Veterinary Medicine, Northwest A&F University, Yangling 712100, China; futingshu@nwafu.edu.cn (T.F.); mengweichen@nwafu.edu.cn (M.C.); panying@nwafu.edu.cn (Y.P.); qin3625@nwafu.edu.cn (X.W.); baixiaonan@nwafu.edu.cn (X.B.); panmenghao@nwafu.edu.cn (M.P.)

**Keywords:** heat stress, taurine, dairy goat, sperm quality, seminal plasma metabolism

## Abstract

Taurine is abundant in seminal plasma and is involved in redox balance, osmoregulation, and sperm membrane stability. However, its role in protecting dairy goat bucks against heat stress-associated declines in sperm quality remains unclear. In this study, eighteen Guanzhong dairy goat bucks were assigned to three groups: control (NC), field heat stress (HS), and HS with taurine supplementation (HS + Tau). Heat stress reduced seminal plasma taurine abundance and was associated with metabolic reprogramming, impaired sperm quality, disturbed redox homeostasis, and decreased LH and testosterone levels. Specifically, HS reduced sperm motility, viability, membrane integrity, and kinematic performance, increased sperm abnormalities, and shortened in vitro sperm survival time. Taurine supplementation alleviated these adverse changes and shifted the seminal plasma metabolome toward a more homeostatic profile. Metabolomic analysis indicated that HS was associated with the accumulation of long-chain acylcarnitines in seminal plasma. Complementary mouse and TM4 Sertoli cell experiments provided preliminary mechanistic support, suggesting that taurine may partially protect Sertoli cell tight-junction proteins, particularly ZO-1, under heat- and acylcarnitine-related stress, and may be associated with the modulation of p38/AKT signaling. Collectively, these findings suggest that taurine alleviates heat stress-induced declines in sperm quality in dairy goat bucks, at least in part, by modulating seminal plasma metabolism.

## Simple Summary

Heat stress reduces sperm quality in dairy goat bucks. In a 60-day on-farm trial, taurine supplementation improved sperm motility, viability, and membrane integrity, reduced abnormalities, and partially restored antioxidant status and reproductive hormones. Metabolomics suggested taurine alleviated heat stress-related metabolic disruption, including long-chain acylcarnitine accumulation. Mouse and TM4 Sertoli cell experiments further indicated taurine helps protect the testicular microenvironment by preserving tight-junction proteins (ZO-1 and occludin) and modulating p38/AKT signaling.

## 1. Introduction

Given the ongoing trend of global climate warming, high-temperature heat stress (HS) has emerged as a pivotal environmental factor significantly impeding the sustainable progress of animal husbandry, particularly impacting ruminants [1]. During the breeding season, dairy goats are frequently exposed to hot and humid environments, rendering them highly vulnerable to HS responses, which can trigger a series of problems including elevated body temperature, endocrine disruption, and metabolic disorders [2]. Research has indicated that HS is associated with metabolic disturbances in seminal plasma, damage to sperm membrane integrity, oxidative damage, and impaired sperm motility and fertilization capacity [3,4,5]. On a tissue level, HS adversely affects testicular function predominantly by triggering oxidative stress, inhibiting steroidogenesis, and disrupting testicular tissue structure [6,7,8]. Collectively, these findings indicate that heat stress is an important threat to male fertility in ruminants, particularly through its adverse effects on sperm quality, endocrine balance, and testicular homeostasis [9,10].

Tight junctions at the blood–testis barrier are essential for maintaining the seminiferous epithelial microenvironment required for spermatogenesis [11,12]. Mild testicular hyperthermia/heat stress disrupts Sertoli cell junctional organization and increases barrier permeability, accompanied by reduced expression and/or mislocalization of TJ proteins such as ZO-1 and occludin, thereby impairing spermatogenic function [13]. Consequently, preserving TJ integrity under HS represents a promising approach to mitigate temperature-induced testicular damage and safeguard male fertility.

In recent years, metabolomics, as a fundamental element of omics technologies, has been widely applied to explore the mechanisms of metabolic reprogramming under complex physiological stress conditions. Compared with single-analyte biochemical assays, metabolomics enables high-throughput, system-level profiling of numerous small-molecule metabolites in tissues and biofluids, thereby capturing dynamic metabolic changes and providing a more comprehensive view of physiological or pathological states [14,15]. Seminal plasma, which serves as the primary microenvironment for sperm survival and function, directly affects sperm membrane stability, mitochondrial function, and redox state through its metabolic composition, ultimately influencing sperm motility and fertilization potential [16,17]. Previous studies have shown that heat stress is associated with a broad shift in the seminal plasma metabolome, including lipid-related pathways and energy metabolism; notably, amino acids and acylcarnitines are abundant seminal plasma metabolites and are closely associated with semen quality parameters [18,19]. Therefore, the analysis of shifts in seminal plasma metabolic profiles under HS utilizing metabolomics, coupled with functional interventions, holds substantial theoretical and practical significance.

Taurine is a sulfur-containing β-amino acid widely distributed in animal tissues, and it is highly abundant in the male reproductive tract, including seminal plasma [20]. It serves crucial functions in osmoregulation, antioxidative stress, membrane stabilization, and energy metabolism regulation [21]. Studies have found that taurine contributes positively to sperm cryopreservation, fertilization capacity, and testicular development [22]. Moreover, taurine has exhibited protective properties in various stress models through reactive oxygen species scavenging, modulation of glutathione metabolism, and inhibition of apoptosis [23,24,25]. Nevertheless, although taurine has been reported to support sperm preservation, redox homeostasis, and male reproductive function, it remains unclear whether taurine can alleviate heat stress-associated declines in sperm quality in dairy goat bucks through the modulation of seminal plasma metabolism, and whether such metabolic effects may be linked to the protection of Sertoli cell junctional function [26,27,28].

Therefore, the present study primarily investigated the effects of taurine supplementation on semen quality and seminal plasma metabolic alterations in dairy goat bucks under summer field conditions. By integrating phenotypic evaluation with untargeted metabolomics, we aimed to determine whether taurine could alleviate heat stress-associated reproductive impairment in goats and to identify the related metabolic changes in seminal plasma. Complementary mouse and TM4 cell experiments were included only to provide preliminary mechanistic support under controlled conditions. This study was designed to address the limited understanding of taurine-associated seminal plasma metabolic remodeling under heat stress and its possible relationship with testicular junctional protection in dairy goat bucks.

## 2. Materials and Methods

### 2.1. Experimental Animals and Grouping

The experimental animals and procedures in this study were carried out in compliance with the Guide for the Care and Use of Laboratory Animals (Ministry of Science and Technology of the People’s Republic of China, Policy No. 2006398). All protocols were reviewed and approved by the Animal Care and Use Committee at Northwest A&F University, Yangling, China (Approval No. 201902A299).

Eighteen clinically healthy adult Guanzhong dairy goat bucks (1.5–2.0 yr old; 70.0 ± 5.0 kg) were enrolled from the same commercial farm (Shaanxi Aonik Dairy Goat Breeding Co., Ltd., Fuping, China). Before the experiment, all bucks were acclimated for 7 d and managed under the same routine conditions, including the same housing environment, basal diet, free access to water, and the same daily husbandry procedures. Animals were randomly assigned (simple randomization) to three groups (*n* = 6 bucks per group): control (NC), heat stress (HS), and heat stress plus taurine (HS + Tau).

The livestock component was conducted as an on-farm summer field study. To minimize seasonal and time-related confounding, all groups were maintained concurrently on the same farm throughout the same 60 d experimental period, and environmental conditions were monitored continuously. During the study, all animals were kept under the same farm management conditions and exposed to the same ambient summer environment. Thus, the only additional intervention applied in the HS + Tau group was taurine supplementation. Taurine (purity ≥ 99%; Sigma-Aldrich (Shanghai) Trading Co., Ltd., Shanghai, China) was administered orally to the HS + Tau group at 100 mg/kg body weight per day, divided into two doses (08:00 and 18:00), throughout the 60 d experimental period. The NC and HS groups underwent the same handling schedule but received no taurine supplementation. The experimental unit was the individual buck.

Semen was collected repeatedly from each buck once every 3 d for 60 d, yielding approximately 20 ejaculates per buck. Repeated ejaculates obtained from the same buck were treated as repeated measures rather than independent biological replicates in the statistical analyses.

### 2.2. Heat Stress Assessment in the On-Farm Study

The goat experiment was conducted as an on-farm summer field study under naturally occurring ambient environmental conditions. During the 60-day experimental period, ambient temperature (T, °C) and relative humidity (RH, %) inside the barn were recorded daily at 13:00 using a digital thermometer–hygrometer (TH101B; Guangzhou Zhaoji Instrument and Meter Manufacturing Co., Ltd., Guangzhou, China). The temperature–humidity index (THI) was calculated accordingly to characterize the environmental heat load conditions experienced by the animals during the study period. THI was calculated as THI = (1.8 × T + 32) − [(0.55 − 0.0055 × RH) × (1.8 × T − 26)] [29]. Thermal load categories were defined as: THI < 72 (no heat stress), 73–77 (mild heat stress), 78–89 (moderate heat stress), and >90 (severe heat stress). In the goat experiment, heat stress was defined by the recorded summer field environment rather than by an experimentally induced heat stress procedure. The recorded temperature, relative humidity, and THI values were used to describe the duration and intensity of environmental heat load exposure during the study period. Rectal temperature was measured using a digital thermometer, and scrotal dimensions were measured using a flexible measuring tape.

### 2.3. Semen Collection and Sperm Quality Evaluation in Bucks

Semen was collected using an artificial vagina (0D40, Beijing Aowotek Livestock Technology Co., Ltd., Beijing, China) in the morning (08:00–09:00). Each buck was collected once every 3 d for 60 d, yielding approximately 20 ejaculates per buck. Immediately after collection, semen samples were maintained at 37 °C and processed within 30 min. Aliquots of each ejaculate were used for sperm quality evaluation and seminal plasma separation. Whenever feasible, technicians performing CASA and staining assessments were blinded to group allocation. Sperm motility, viability, and motion characteristics, including total motility and kinematic parameters, were measured using a computer-assisted sperm analysis system (CASA; KD-306, Taian Kede, Taian, China) according to the manufacturer-recommended settings for goat semen. Sperm plasma membrane integrity was evaluated using the hypo-osmotic swelling test (HOST). Briefly, 1 mL of hypo-osmotic solution was added to a 1.5 mL microcentrifuge tube and prewarmed at 37 °C for 5 min. Then, 100 μL of semen was added and mixed gently. After incubation at 37 °C for 30 min, 10 μL of the mixture was placed on a glass slide, covered with a coverslip, and examined under a phase-contrast microscope. A total of 200 spermatozoa were counted per sample, and sperm showing tail swelling were considered to have normal plasma membrane function. The percentage of sperm with intact plasma membranes was calculated as follows: sperm membrane integrity (%) = number of sperm with swollen tails/total number of counted sperm × 100%. Acrosome integrity was evaluated by Giemsa staining. Briefly, semen smears were prepared on glass slides, air-dried, fixed, and stained with Giemsa solution. After staining, sperm were observed under a light microscope, and at least 200 spermatozoa were counted per sample. Sperm with intact and uniformly stained acrosomal regions were considered acrosome-intact, and the percentage of acrosome-intact sperm was calculated accordingly. Sperm morphological abnormalities were evaluated using crystal violet staining. After staining, sperm morphology was examined under a microscope, and at least 200 spermatozoa were counted per slide. Two slides were prepared for each ejaculate, and the sperm abnormality rate was calculated as the percentage of morphologically abnormal sperm among the total counted sperm.

#### Chilled Storage Test

To evaluate whether taurine directly influences semen preservation, semen was diluted (1:4, *v*/*v*) with either (i) a conventional extender (0.685 g glucose, 0.685 g fructose, 1.6 g citric acid, 3.025 g Tris, 0.1 g EDTA, and 200,000 IU penicillin in ultrapure water to 100 mL; supplemented with 10% fresh egg yolk before use) or (ii) the same extender supplemented with taurine to a final concentration of 10 mM. Diluted semen was stored at 4 °C, and motility and viability were assessed daily after rewarming until no viable sperm were detected. To determine the appropriate working concentration of taurine for semen preservation during chilled storage, freshly collected semen was diluted 1:4 (*v*/*v*) with the basal extender and supplemented with different concentrations of taurine. The diluted semen samples were then stored at 4 °C. On day 2 of chilled storage, samples were rewarmed before analysis, and sperm motility was evaluated. The concentration showing the best protective effect on sperm motility was selected as the working concentration. Based on this screening experiment, 10 mM taurine was used in the subsequent chilled storage assay to evaluate sperm survivability during liquid storage.

### 2.4. Seminal Plasma Preparation and Untargeted Metabolomics

Seminal plasma was separated by centrifugation (7000× *g*, 15 min, 4 °C), and the supernatant was aliquoted and stored at −80 °C until analysis. All seminal plasma collected within 60 days was pooled according to the experimental groups (NC, HS, HS + Tau), resulting in three seminal plasma samples. Each sample was subjected to metabolomic and biochemical analyses, with three or more repeated measurements. Untargeted metabolomics was conducted using UHPLC coupled to quadrupole time-of-flight mass spectrometry (UHPLC-QTOF/MS) in both positive and negative ion modes (Wuhan MetWare Biotechnology Co., Ltd., Wuhan, China). Samples were extracted with cold methanol/acetonitrile containing internal standards, vortexed, sonicated, incubated at −20 °C, and centrifuged to remove precipitated proteins. Pooled quality control (QC) samples were prepared by combining equal aliquots from each extract and were injected periodically to monitor instrument stability. Raw data were processed for peak detection, alignment, and deconvolution using GNPS_Vendor_Conversion software (version 5.0) or XCMS (version 4.4.0). Features detected in <50% of samples within a group were removed; remaining missing values were imputed with a small value (half of the minimum positive value) before log transformation. Features with poor QC reproducibility (QC RSD > 30%) were excluded. Data were normalized to internal standards and total ion signal and Pareto-scaled prior to multivariate analyses. Unsupervised principal component analysis (PCA) was used for outlier inspection. Supervised partial least squares discriminant analysis (PLS-DA) was used for group separation and was validated by k-fold cross-validation and permutation testing (≥200 permutations). Differential metabolites were defined by variable importance in projection (VIP > 1) together with false discovery rate control (Benjamini–Hochberg q < 0.05) and an absolute fold change threshold (|log2FC| ≥ 1, unless otherwise stated). Metabolite annotation and pathway enrichment were performed using KEGG and HMDB.

### 2.5. Testosterone, Total Acylcarnitines, and Oxidative Stress Indices in Goat Seminal Plasma

To assess endocrine and redox changes associated with heat load and taurine supplementation, we analyzed testosterone, total acylcarnitines, and oxidative stress indices in goat seminal plasma. For these assays, semen collected across the experimental period was centrifuged to obtain seminal plasma, and aliquots were pooled by treatment group to generate one composite sample per group.

Testosterone and total acylcarnitines were quantified using commercial ELISA kits (Shanghai Fankewei Biotechnology Co., Ltd., Shanghai, China). Reactive oxygen species (ROS), malondialdehyde (MDA), superoxide dismutase (SOD) activity, and glutathione (GSH) content were measured using commercial assay kits (Nanjing Jiancheng Bioengineering Institute, Nanjing, China). All assays were performed according to the manufacturers’ instructions and were based on the standard HRP-TMB colorimetric system. Briefly, standards and appropriately prepared seminal plasma samples were added to the designated wells of the microplate and incubated under the prescribed conditions. After incubation, the wells were washed, followed by the addition of the HRP-conjugated reagent. After a further incubation and washing step, TMB substrate was added for color development. The reaction was terminated with stop solution, and the absorbance was measured at 450 nm using a microplate reader. The concentrations or activities of testosterone, total acylcarnitines, ROS, MDA, SOD, and GSH were calculated according to the corresponding standard curves provided with the assay kits. All assays were run in technical duplicate, and the intra-assay coefficients of variation were <10%.

### 2.6. Mouse Model of Heat Stress and Taurine Treatment

All mouse protocols were reviewed and approved by the Animal Ethical and Welfare Committee of Northwest A&F University (Approval No. 201902A299). To provide controlled in vivo validation, a murine heat stress model was established using 8-week-old male Kunming mice (30–35 g). Mice were acclimatized for 7 d under controlled conditions (23 ± 2 °C, 50 ± 10% humidity, 12 h light/12 h dark cycle) with free access to food and water and were randomly assigned to NC, HS, and HS + Tau groups (*n* = 10 per group). Mice in the HS and HS + Tau groups were exposed to 37 °C for 2 h/day at noon for 45 consecutive days. The HS + Tau group received 0.1% (*w*/*v*) taurine as the sole drinking water source, replaced daily, for 45 days. NC mice were maintained under standard room conditions (23 ± 2 °C, 50 ± 10% relative humidity, 12 h light/12 h dark cycle) without additional heat exposure or taurine treatment. At the end of exposure, testes, epididymides, and blood were collected for downstream analyses. Epididymal sperm were assessed for motility, morphology (crystal violet staining), and daily sperm production as described below.

### 2.7. In Vitro Fertilization and Zona Pellucida Binding Assay

In vitro fertilization (IVF) and zona pellucida binding assays were performed using epididymal sperm from the mouse model. Cumulus–oocyte complexes were obtained from superovulated female mice and handled in TYH medium. Epididymal sperm were collected from individual males, capacitated in TYH medium, and used for IVF without pooling across males. Each IVF session used sperm from a single male and oocytes from one or more female donors. Cleavage rate (2-cell embryos) and subsequent development were recorded under standard culture conditions. For zona pellucida binding, zona-intact oocytes were co-incubated with capacitated sperm for a defined period, gently washed, and the number of sperm bound per oocyte was counted under microscopy by an assessor blinded to treatment. For embryo outcome analysis, the male was the primary unit of analysis. Data from oocytes and embryos were treated as observations nested within each male and donor. Embryo proportions were analyzed using a generalized linear mixed model with a binomial distribution. In this model, treatment was included as a fixed effect, while male and donor were included as random effects to account for within-group correlations.

### 2.8. TM4 Cell Heat Stress Model and Treatments

Mouse Sertoli TM4 cells (provided by the Laboratory of Animal and Embryo Engineering, Northwest A&F University) were cultured in high-glucose DMEM/F-12 containing 10% fetal bovine serum and 1% penicillin–streptomycin at 37 °C with 5% CO_2_. Cells were routinely tested for mycoplasma contamination and were used at low passage numbers. For heat stress (HS), cells were incubated at 42.5 °C for 1 h and then returned to 37 °C for 6 h of recovery. This condition was selected with reference to previously reported in vitro heat stress models [30,31,32]. Taurine pretreatment (5, 10, 20, 30, and 40 mM) was applied for 6 h prior to HS. The working taurine concentration for subsequent experiments was selected based on CCK-8 viability screening. For lipid/metabolic stress, cells were treated with L-carnitine-related acylcarnitine across a dose range, with or without taurine, and then harvested for protein analysis.

### 2.9. Western Blot

Testicular tissues and TM4 cells were lysed in RIPA buffer supplemented with protease and phosphatase inhibitors. Protein concentrations were determined by BCA assay, and equal amounts of protein were separated by SDS–PAGE and transferred to PVDF membranes. Membranes were blocked and incubated overnight at 4 °C with primary antibodies against ZO-1, occludin, p38, phospho-p38, AKT, phospho-AKT, and β-actin, followed by HRP-conjugated secondary antibodies. Protein bands were detected by enhanced chemiluminescence and imaged using a digital imaging system under non-saturating conditions. Densitometry was performed in ImageJ (version 1.54g). ZO-1 and occludin signals were normalized to β-actin; phospho/total ratios were calculated for p38 and AKT. For figure preparation, linear adjustments (brightness/contrast) were applied uniformly to the entire image when needed, and no features were enhanced or removed.

### 2.10. Immunofluorescence Staining

Immunofluorescence staining was used to assess the localization and relative abundance of ZO-1 and occludin in mouse testicular sections and TM4 cells. Paraffin sections were deparaffinized and rehydrated; TM4 cells were fixed with 4% paraformaldehyde. After antigen retrieval (sections) and blocking with 5% BSA, samples were incubated with primary antibodies followed by fluorophore-conjugated secondary antibodies. Nuclei were counterstained with DAPI. Images were acquired using a laser scanning confocal microscope with identical acquisition settings for group comparisons. Representative images are shown with scale bars. Image processing was limited to linear adjustments applied equally across groups.

### 2.11. Histological Examination of Mouse Testicular Tissue

Mouse testes were fixed in 4% paraformaldehyde for 24 h, dehydrated, paraffin-embedded, and sectioned for hematoxylin–eosin (H&E) staining following standard procedures. Five random fields per section were captured for analysis. Seminiferous tubule diameter, lumen diameter, and seminiferous epithelium thickness were quantified using ImageJ by an assessor blinded to treatment.

### 2.12. Validation and Quality Assurance

Animals were allocated to groups by simple randomization. Semen collection and CASA settings were standardized and performed by trained personnel using the same equipment and operating procedures throughout the study. For UHPLC-QTOF/MS metabolomics, internal standards were included during extraction and pooled QC samples were injected periodically to monitor instrument stability; data were inspected using PCA for outliers. For microscopy and Western blotting, imaging and acquisition settings were kept constant across groups, and analyses were performed using the same processing pipeline for all samples. Cell culture experiments were performed using consistent passage ranges and included independent biological replicates.

### 2.13. Statistical Analysis

Statistical analyses were performed in R (version 4.5.1) and SAS (v9.0). Prior to modeling, distributions and residual diagnostics (including Q–Q plots and residual-versus-fitted plots) were inspected; potential outliers were examined and were not excluded unless justified a priori. The experimental unit was the individual animal (buck or mouse) or the independent cell culture replicate, as appropriate. For the goat study, semen traits measured repeatedly across ejaculates were analyzed using linear mixed-effects models with treatment group as a fixed effect and buck as a random effect; within-buck repeated measures were modeled using an appropriate covariance structure specified a priori based on the repeated-measures design. Least squares means were compared with Tukey adjustment. For goat seminal plasma endpoints (biochemical assays) and other single-time-point outcomes, treatment effects were evaluated by one-way ANOVA followed by Tukey’s test, or by Kruskal–Wallis followed by Dunn’s test when distributional assumptions were not met. For mouse endpoints, treatment effects were evaluated by one-way ANOVA with Tukey’s multiple comparison test or by nonparametric alternatives when required. Embryo development outcomes were analyzed using generalized linear mixed models (binomial distribution) with treatment as a fixed effect and male and donor as random effects to account for nesting. For untargeted metabolomics, univariate testing was performed at the feature/metabolite level and multiplicity was controlled using the Benjamini–Hochberg procedure (q < 0.05). Because metabolomics used one pooled seminal plasma sample per group (with technical replicate injections), metabolomics results are interpreted as exploratory and hypothesis-generating. Supervised multivariate models (PLS-DA) were reported together with cross-validation and permutation test results. Data are presented as mean ± SD unless otherwise stated; repeated-measures outcomes are presented as model-based estimates (least squares means) with associated uncertainty (SEM or 95% CI) as indicated in figure legends. Statistical significance was set at *p* < 0.05 (two-sided). Analysis scripts and additional quality control details are provided in the Supplementary Information.

## 3. Results

### 3.1. Field Heat Stress Induces Physiological Responses and Testicular Changes in Bucks

**Figure 1:**
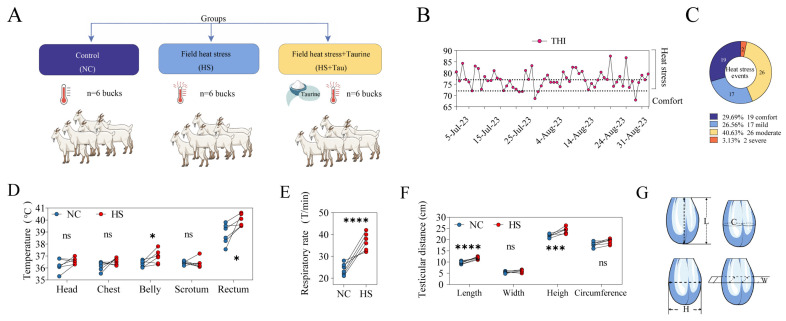
Field heat stress exposure and physiological and testicular phenotypes in bucks. (**A**) Group allocation of bucks: control (NC), field heat stress (HS), and field heat stress plus taurine (HS + Tau) (*n* = 6 bucks/group); Colors indicate different groups: deep purple, NC group; light blue, HS group; and light yellow, HS + Tau group. (**B**) Daily temperature–humidity index (THI) during the experimental period; dashed lines indicate the thresholds separating comfort and heat stress conditions. (**C**) Distribution of recorded heat stress events across thermal load categories (comfort, mild, moderate, severe). (**D**) Surface temperatures at the head, chest, belly, and scrotum, and rectal temperature in bucks; “ns” indicates no significant difference between groups. (**E**) Respiratory rate (times/min) in bucks. (**F**) Testicular/scrotal morphometric measurements including testicular length, width, height, and scrotal circumference (cm). (**G**) Schematic illustration of measurement positions/definitions: L, testicular length; W, testicular width; H, testicular height; C, scrotal circumference. Data are presented as mean ± SD with individual values shown. Statistical significance is denoted as * *p* < 0.05, *** *p* < 0.001, **** *p* < 0.0001; ns, *p* ≥ 0.05.

Heat load and physiological indices were recorded to verify that bucks experienced measurable HS during the experimental period and to describe corresponding testicular phenotypic responses (Figure 1). Bucks were randomly assigned to NC, HS, or HS + Tau (*n* = 6/group) (Figure 1A).

Environmental monitoring showed that the temperature–humidity index (THI) remained elevated for substantial portions of the study and repeatedly exceeded the heat stress threshold indicated in the figure (Figure 1B). When thermal load events were categorized by severity (Figure 1C), a total of 64 events were recorded, including 19 comfort events (29.69%), 17 mild events (26.56%), 26 moderate events (40.63%), and two severe events (3.13%), indicating frequent heat load exposure dominated by mild-to-moderate conditions.

Consistent with heat load exposure, HS bucks exhibited higher belly surface temperature (*p* < 0.05) and higher rectal temperature (*p* < 0.05) compared with NC (Figure 1D), whereas surface temperatures at the head, chest, and scrotum did not differ among groups (*p* > 0.05) (Figure 1D). Respiratory rate was markedly increased under HS conditions (*p* < 0.001) (Figure 1E). For testicular and scrotal morphometrics, testicular length was greater in HS than in NC (*p* < 0.001) and testicular height was also higher in HS (*p* < 0.01) (Figure 1F), while testicular width and scrotal circumference were not different among groups (*p* > 0.05) (Figure 1F). Measurement locations and definitions (L, length; W, width; H, height; C, scrotal circumference) are illustrated in Figure 1G.

Overall, these measurements indicate sustained heat load during the study, accompanied by increases in rectal temperature and respiratory rate (Figure 1D,E) and by changes in selected testicular morphometrics (Figure 1F,G).

### 3.2. Taurine Supplementation Improves Sperm Quality and Redox Endocrine Status in Heat-Stressed Bucks

**Figure 2:**
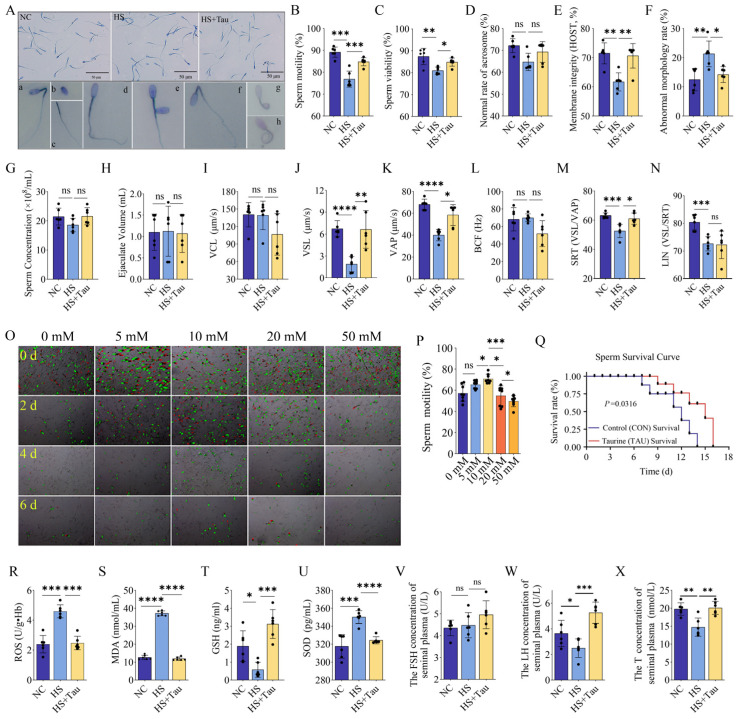
Taurine supplementation is associated with improved semen quality, sperm function, oxidative stress indices, and hormone levels in heat-stressed bucks. (**A**) Representative sperm smear images from NC, HS, and HS + Tau groups (scale bar = 50 μm) and examples of abnormal sperm morphology (**a**–**h**). (**B**–**F**) Sperm quality parameters: sperm motility (**B**), sperm viability (**C**), normal acrosome rate (**D**), membrane integrity assessed by HOST (**E**), and abnormal morphology rate (**F**). (**G**,**H**) Semen quality traits: sperm concentration (**G**) and ejaculate volume (**H**). (**I**–**N**) CASA-derived kinematic parameters: VCL (**I**), VSL (**J**), VAP (**K**), BCF (**L**), STR (**M**), and LIN (**N**). (**O**) Representative computer-assisted sperm analysis (CASA) images showing sperm motility under different taurine concentrations. Live sperm are indicated in red, and motility trajectories are shown in green. (**P**) Sperm motility significantly increased following treatment with 10 mM taurine. (**Q**) Sperm survival was extended to 16 days in the presence of 10 mM taurine. (**R**–**U**) Oxidative stress indices in seminal plasma: ROS (**R**), MDA (**S**), GSH (**T**), and SOD (**U**). (**V**–**X**) Hormone concentrations in seminal plasma: testosterone (**V**), LH (**W**), and FSH (**X**). Panels (**B**–**N**) were derived from repeated semen collections from 6 bucks per group during the experimental period, and repeated ejaculates from the same buck were treated as repeated measures in the statistical analysis. Panels (**R**–**X**) show biochemical and hormonal indices measured from individual seminal plasma samples (*n* = 6 per group), with all assays performed in technical duplicate. Data are presented as mean ± SD with individual values shown. Statistical significance is denoted as * *p* < 0.05, ** *p* < 0.01, *** *p* < 0.001, **** *p* < 0.0001; ns, *p* ≥ 0.05. Black dots indicate individual data points.

To evaluate the effects of field heat stress and taurine supplementation on semen quality and sperm function, we assessed sperm morphology and functional traits, CASA-derived kinematic parameters, the in vitro response to taurine, and seminal plasma oxidative stress indices and reproductive hormones (Figure 2). Representative sperm smear images and examples of abnormal morphology are shown in Figure 2A.

Heat stress was associated with reduced sperm quality. Sperm motility was lower in the HS group than in NC (*p* < 0.001), whereas motility was higher in HS + Tau than in HS (*p* < 0.001); HS + Tau also differed from NC (*p* < 0.01) (Figure 2B). Sperm viability was lower in HS than in NC (*p* < 0.01) and higher in HS + Tau than in HS (*p* < 0.05), with no difference between HS + Tau and NC (*p* > 0.05) (Figure 2C). Acrosome integrity did not differ among groups (*p* > 0.05) (Figure 2D). Plasma membrane integrity was reduced in HS compared with NC (*p* < 0.01) and increased in HS + Tau compared with HS (*p* < 0.01), with no difference between HS + Tau and NC (*p* > 0.05) (Figure 2E). The abnormal morphology rate was higher in HS than in NC (*p* < 0.01) and lower in HS + Tau than in HS (*p* < 0.05), with no difference between HS + Tau and NC (*p* > 0.05) (Figure 2F). Semen concentration and ejaculate volume did not differ among groups (both *p* > 0.05) (Figure 2G,H).

CASA analyses further indicated altered sperm kinematics under heat stress. VCL did not differ among groups (*p* > 0.05) (Figure 2I). VSL was lower in HS than in NC (*p* < 0.0001) and higher in HS + Tau than in HS (*p* < 0.01), with no difference between HS + Tau and NC (*p* > 0.05) (Figure 2J). Similarly, VAP was lower in HS than in NC (*p* < 0.0001) and higher in HS + Tau than in HS (*p* < 0.05), with no difference between HS + Tau and NC (*p* > 0.05) (Figure 2K). BCF did not differ among groups (*p* > 0.05) (Figure 2L). STR was lower in HS than in NC (*p* < 0.001) and higher in HS + Tau than in HS (*p* < 0.05), with no difference between HS + Tau and NC (*p* > 0.05) (Figure 2M). LIN was lower in HS than in NC (*p* < 0.001); HS and HS + Tau did not differ (*p* > 0.05), whereas HS + Tau remained lower than NC (*p* < 0.01) (Figure 2N).

To investigate the protective effect of taurine on sperm during chilled storage, we evaluated the impact of different taurine concentrations on sperm motility using a computer-assisted sperm analysis (CASA) system and further examined the effect of the optimal protective concentration (10 mM) on sperm survival duration. As shown in Figure 2O, live sperm are indicated in red, and motility trajectories are shown in green. The results demonstrate that 10 mM taurine significantly enhanced sperm motility (Figure 2P) and extended sperm survival to 16 days (Figure 2Q).

Seminal plasma redox indices were also altered by heat stress and taurine supplementation. ROS was higher in HS than in NC (*p* < 0.001) and lower in HS + Tau than in HS (*p* < 0.001), with no difference between HS + Tau and NC (*p* > 0.05) (Figure 2R). MDA was higher in HS than in NC (*p* < 0.0001) and lower in HS + Tau than in HS (*p* < 0.0001), with no difference between HS + Tau and NC (*p* > 0.05) (Figure 2S). GSH was lower in HS than in NC (*p* < 0.05) and higher in HS + Tau than in HS (*p* < 0.001), with no difference between HS + Tau and NC (*p* > 0.05) (Figure 2T). SOD activity was higher in HS than in NC (*p* < 0.001) and lower in HS + Tau than in HS (*p* < 0.0001), with no significant difference between HS + Tau and NC (*p* > 0.05) (Figure 2U). For reproductive hormones, testosterone was lower in HS than in NC (*p* < 0.01) and higher in HS + Tau than in HS (*p* < 0.01), with no difference between HS + Tau and NC (*p* > 0.05) (Figure 2V). LH was lower in HS than in NC (*p* < 0.05) and higher in HS + Tau than in HS (*p* < 0.001), with no difference between HS + Tau and NC (*p* > 0.05) (Figure 2W). FSH did not differ among groups (*p* > 0.05) (Figure 2X).

### 3.3. Untargeted Metabolomics Reveals Heat Stress-Related Seminal Plasma Metabolic Reprogramming and Taurine-Associated Shifts

**Figure 3:**
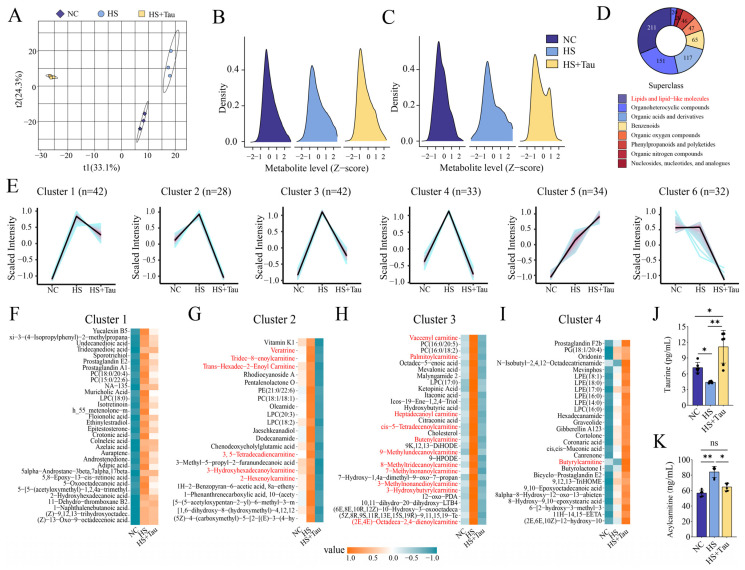
Untargeted metabolomics reveals heat stress-associated seminal plasma metabolic alterations and taurine-related shifts. (**A**) Multivariate score plot showing separation of NC, HS, and HS + Tau groups based on the global seminal plasma metabolome; Colors indicate different groups: deep purple dot, NC group; light blue dot, HS group; and light yellow dot, HS + Tau group (**B**,**C**) Density distributions of normalized metabolite abundances (Z-scores) across groups in two metabolomics datasets/modes. (**D**) Superclass annotation of differential metabolites among groups. (**E**) Trend clustering of differential metabolites across NC, HS, and HS + Tau, showing six representative abundance patterns (cluster size indicated in each panel). (**F**–**I**) Heatmaps displaying representative metabolites within clusters 1–4 (scaled values). (**J**) Relative abundance of taurine in seminal plasma. (**K**) Relative abundance of acylcarnitine in seminal plasma. Untargeted metabolomics was conducted using pooled seminal plasma samples. Data are presented as mean ± SD with individual values shown where applicable. Statistical significance is denoted as * *p* < 0.05, ** *p* < 0.01; ns, *p* ≥ 0.05. Black dots indicate individual data points.

To further characterize the biochemical milieu accompanying the observed changes in sperm quality, we profiled the seminal plasma metabolome of NC, HS, and HS + Tau bucks using untargeted metabolomics (Figure 3). Multivariate analysis showed separation among the three groups, indicating group-associated differences in global metabolomic profiles (Figure 3A). After Z-score normalization, the overall distributions of metabolite abundances differed across groups, and similar between-group distributional patterns were observed in both datasets/modes (Figure 3B,C), suggesting that group differences were not driven by a small number of extreme features. Differential metabolites were then classified by chemical superclass. Lipids and lipid-like molecules accounted for the largest proportion of differential metabolites, followed by heterocyclic compounds, organic acids and derivatives, and benzenoids (Figure 3D). To summarize coordinated changes across groups, differential metabolites were clustered by their abundance trajectories across NC, HS, and HS + Tau. This analysis identified six representative trend patterns (Figure 3E). Heatmaps of representative clusters illustrated within-cluster concordance, with metabolites showing coordinated shifts across groups (Figure 3F–I; Table S1), consistent with systematic remodeling of the seminal plasma metabolome under heat stress and a distinct metabolomic pattern associated with taurine supplementation. At the level of selected metabolites, seminal plasma taurine abundance differed among groups. Taurine was lower in HS than in NC (*p* < 0.05) and higher in HS + Tau than in HS (*p* < 0.01); HS + Tau also exceeded NC (*p* < 0.05) (Figure 3J). In contrast, acetylcarnitine was higher in HS than in NC (*p* < 0.01) and lower in HS + ImaTau than in HS (*p* < 0.05), with no difference between HS + Tau and NC (*p* > 0.05) (Figure 3K). Collectively, these metabolomics data indicate that heat stress is associated with broad changes in seminal plasma metabolites, with differential features enriched for lipid-related classes, and that taurine supplementation is accompanied by increased seminal plasma taurine and shifts in selected metabolites including acetylcarnitine (Figure 3).

### 3.4. Heat Stress Reduces Testicular Seminiferous Epithelial Thickness and Sertoli Tight-Junction Proteins in Mice and Taurine Alleviates These Effects

**Figure 4:**
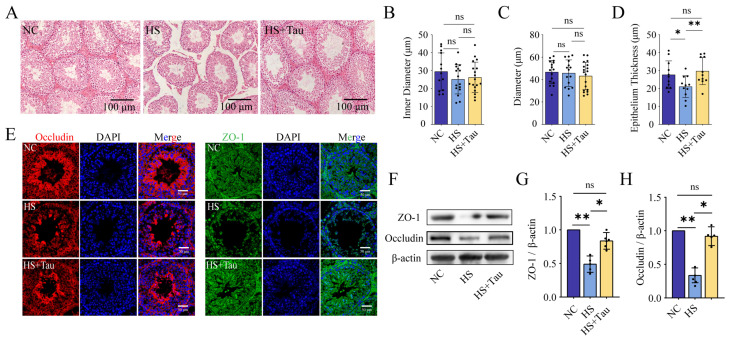
Taurine treatment is associated with changes in testicular histology and tight-junction proteins in the mouse heat stress model. (**A**) Representative H&E-stained testis sections from NC, HS, and HS + Tau mice (scale bar = 100 μm). (**B**–**D**) Quantification of seminiferous tubule structure: inner diameter (**B**), tubule diameter (**C**), and seminiferous epithelium thickness (**D**). (**E**) Immunofluorescence staining of occludin (red) and ZO-1 (green) in seminiferous tubules with DAPI (blue) (scale bar = 50 μm). (**F**) Representative Western blots of ZO-1 and occludin, with β-actin used as the loading control (*n* = 4 independent biological replicates). (**G**,**H**) Densitometric quantification of ZO-1 (**G**) and occludin (**H**), normalized to β-actin. Data are presented as mean ± SD with individual values shown where applicable. Statistical significance is denoted as * *p* < 0.05, ** *p* < 0.01; ns, *p* ≥ 0.05. Black dots indicate individual data points.

To examine whether heat stress is accompanied by changes in testicular architecture and seminiferous tubule tight junctions under controlled conditions, we evaluated mouse testes by H&E staining and quantified seminiferous tubule morphometrics, and we assessed Occludin and ZO-1 localization and protein abundance (Figure 4). Representative H&E images are shown in Figure 4A.

Quantitative morphometric analysis showed no differences among groups in seminiferous tubule inner diameter or tubule diameter (*p* > 0.05) (Figure 4B,C). In contrast, seminiferous epithelial thickness differed among groups: epithelial thickness was lower in HS than in NC (*p* < 0.05) and higher in HS + Tau than in HS (*p* < 0.01), with no difference between HS + Tau and NC (*p* > 0.05) (Figure 4D).

Immunofluorescence staining indicated a peripheral, ring-like distribution of occludin and ZO-1 around seminiferous tubules (Figure 4E). Compared with NC, signal intensity appeared reduced and discontinuous in HS, whereas signal intensity and continuity were greater in HS + Tau than in HS (Figure 4E). Western blot results were consistent with these observations (Figure 4F). Densitometric quantification showed lower ZO-1 abundance in HS than in NC (*p* < 0.01) and higher ZO-1 in HS + Tau than in HS (*p* < 0.05), with no difference between HS + Tau and NC (*p* > 0.05) (Figure 4G). Occludin abundance was lower in HS than in NC (*p* < 0.01) and higher in HS + Tau than in HS (*p* < 0.05), with no difference between HS + Tau and NC (*p* > 0.05) (Figure 4H).

Together, these data indicate that heat stress is associated with reduced seminiferous epithelial thickness and decreased expression of the tight-junction proteins ZO-1 and occludin, and that these measures differ under taurine treatment (Figure 4).

### 3.5. Heat Stress Impairs Sperm Fertilization-Related Functions and Embryo Cleavage in Mice and Taurine Improves Outcomes

**Figure 5:**
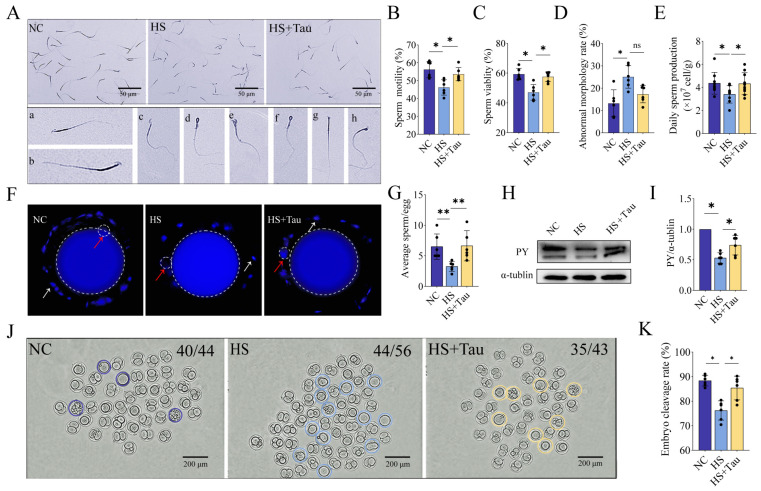
Taurine treatment is associated with improved sperm fertilization-related outcomes in the mouse heat stress model. (**A**) Representative sperm smear images from NC, HS, and HS + Tau mice (scale bar = 50 μm) and examples of abnormal sperm morphology (**a**–**h**). (**B**–**E**) Sperm functional traits: sperm motility (**B**), sperm viability (**C**), abnormal morphology rate (**D**), and daily sperm production (**E**). (**F**) Representative images of the zona pellucida binding assay; dashed circle indicates the oocyte; red arrows indicate bound sperm, and white arrows indicate sperm bound to the oocyte/zona pellucida. (**G**) Quantification of sperm bound per oocyte (average sperm/egg). (**H**) Representative Western blot of sperm protein tyrosine phosphorylation (PY), with α-tubulin serving as the loading control (*n* = 4 independent biological replicates). (**I**) Quantification of PY normalized to α-tubulin. (**J**) Representative images of embryos after IVF (numbers indicate cleaved/total); colored circles indicate uncleaved embryos, with purple for the NC group, blue for the HS group, and yellow for the HS + Tau group; scale bar = 200 μm. (**K**) Embryo cleavage rate (%). Data are presented as mean ± SD with individual values shown where applicable. Statistical significance is denoted as * *p* < 0.05, ** *p* < 0.01; ns, *p* ≥ 0.05. Black dots indicate individual data points.

To evaluate fertilization-related sperm functions and downstream developmental outcomes under controlled conditions, we assessed sperm quality traits, zona pellucida binding capacity, sperm protein tyrosine phosphorylation, and embryo cleavage after IVF in the mouse model (Figure 5). Representative sperm smear images and examples of abnormal morphology are shown in Figure 5A.

For baseline sperm traits, motility was lower in HS than in NC (*p* < 0.05) and higher in HS + Tau than in HS (*p* < 0.05), with no difference between HS + Tau and NC (*p* > 0.05) (Figure 5B). Viability was lower in HS than in NC (*p* < 0.05) and higher in HS + Tau than in HS (*p* < 0.05), with no difference between HS + Tau and NC (*p* > 0.05) (Figure 5C). The abnormal morphology rate was higher in HS than in NC (*p* < 0.05), whereas HS + Tau did not differ from HS (*p* > 0.05) (Figure 5D). Daily sperm production was lower in HS than in NC (*p* < 0.05) and higher in HS + Tau than in HS (*p* < 0.05), with no difference between HS + Tau and NC (*p* > 0.05) (Figure 5E).

Representative images from the zona pellucida binding assay are shown in Figure 5F. Quantification showed fewer sperm bound per oocyte in HS than in NC (*p* < 0.01) and more sperm bound per oocyte in HS + Tau than in HS (*p* < 0.01), with no difference between HS + Tau and NC (*p* > 0.05) (Figure 5G).

Protein tyrosine phosphorylation (PY) patterns are shown in Figure 5H. Densitometric analysis indicated lower PY/α-tubulin in HS than in NC (*p* < 0.05) and higher PY/α-tubulin in HS + Tau than in HS (*p* < 0.05), with no difference between HS + Tau and NC (*p* > 0.05) (Figure 5I).

Representative embryo images following IVF are shown in Figure 5J. The embryo cleavage rate was lower in HS than in NC (*p* < 0.05) and higher in HS + Tau than in HS (*p* < 0.05), with no difference between HS + Tau and NC (*p* > 0.05) (Figure 5K).

### 3.6. Long-Chain Acylcarnitines Reduce Tight-Junction Proteins in TM4 Sertoli Cells and Taurine Restores Expression

**Figure 6:**
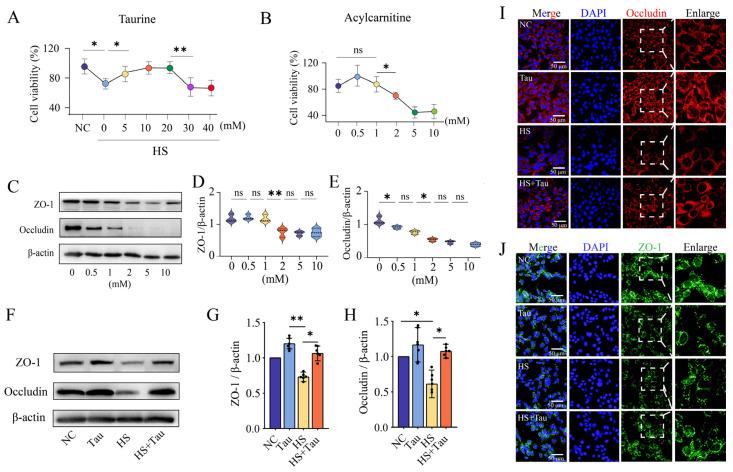
Taurine and acylcarnitine are associated with Sertoli cell viability and tight-junction protein expression in vitro and in vivo. (**A**) TM4 Sertoli cell viability (%) under heat stress (HS) conditions with graded taurine concentrations; NC indicates the thermoneutral control condition; different dot colors indicate the added taurine concentrations. (**B**) TM4 cell viability (%) under graded acylcarnitine concentrations; different circle colors indicate the added acylcarnitine concentrations. (**C**) Representative Western blots of ZO-1 and occludin in TM4 cells treated with increasing concentrations of acylcarnitine, with β-actin serving as the loading control (*n* = 4 independent biological replicates). (**D**,**E**) Quantification of ZO-1 (**D**) and occludin (**E**), normalized to β-actin, across acylcarnitine doses. (**F**) Representative Western blots of ZO-1 and occludin in mouse testes from NC, Tau, HS, and HS + Tau groups; β-actin as loading control (*n* = 4 independent biological replicates). (**G**,**H**) Densitometric quantification of ZO-1 (**G**) and occludin (**H**), normalized to β-actin; black dots indicate individual data points. (**I**,**J**) Immunofluorescence staining of occludin (**I**) red and ZO-1 (**J**) green in mouse testis sections with DAPI (blue); dashed boxes indicate regions enlarged on the right (scale bar = 50 μm). Data are presented as mean ± SD with individual values shown where applicable. Statistical significance is denoted as * *p* < 0.05, ** *p* < 0.01; ns, *p* ≥ 0.05. Abbreviations: HS, heat stress; Tau, taurine.

To evaluate the association between acylcarnitine-related metabolic stress and tight-junction protein expression, we examined TM4 Sertoli cells and mouse testes for ZO-1 and occludin expression under the indicated treatments (Figure 6). In TM4 cells under heat stress conditions, cell viability differed across taurine concentrations (Figure 6A). Viability in the 0 mM taurine group was lower than NC (*p* < 0.05), viability at 5 mM was higher than 0 mM (*p* < 0.05), and viability at 30 mM was lower than 20 mM (*p* < 0.01) (Figure 6A). Under acylcarnitine exposure, 0 mM and 1 mM did not differ (*p* > 0.05), whereas 1 mM differed from 2 mM (*p* < 0.05), with further reductions at higher doses (Figure 6B). Western blots of ZO-1 and occludin after graded acylcarnitine exposure are shown in Figure 6C. Densitometric quantification showed a difference in ZO-1 abundance between 1 mM and 2 mM (*p* < 0.01) (Figure 6D). For occludin, differences were observed between 0 mM and 0.5 mM (*p* < 0.05) and between 1 mM and 2 mM (*p* < 0.05) (Figure 6E). In mouse testes, representative Western blots for ZO-1 and occludin are shown in Figure 6F. ZO-1 abundance was higher in Tau than in HS (*p* < 0.01) and higher in HS + Tau than in HS (*p* < 0.05) (Figure 6G). Occludin abundance was higher in Tau than in NC (*p* < 0.05) and higher in HS + Tau than in HS (*p* < 0.05) (Figure 6H). Immunofluorescence staining further showed ring-like localization of occludin and ZO-1 at the periphery of seminiferous tubules (Figure 6I,J), with weaker and more discontinuous signals in HS and greater signal intensity and continuity in HS + Tau (Figure 6I,J).

### 3.7. Heat Stress and Long-Chain Acylcarnitines Alter Tight-Junction Proteins and p38 and Akt Signaling in TM4 Sertoli Cells

**Figure 7:**
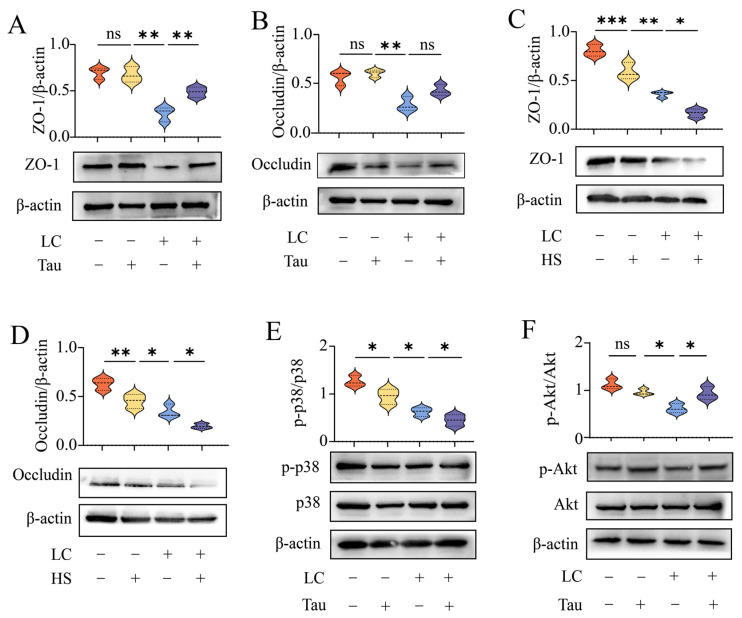
Acylcarnitine and heat stress are associated with p38 and Akt phosphorylation and tight-junction protein expression in TM4 cells. (**A**,**B**) ZO-1 (**A**) and occludin (**B**) protein levels in TM4 cells treated with acylcarnitine (LC) and/or taurine (Tau), with representative blots shown below each panel; values normalized to β-actin. (**C**,**D**) ZO-1 (**C**) and occludin (**D**) protein levels in TM4 cells under LC and/or HS conditions, with representative blots shown below each panel; values normalized to β-actin. (**E**) p-p38/p38 ratio under LC and/or Tau conditions, with representative blots shown below. (**F**) p-Akt/Akt ratio under LC and/or Tau conditions, with representative blots shown below. All Western blot data were derived from four independent biological replicates (*n* = 4). Treatment combinations (LC, Tau, HS) are indicated under each blot. Data are presented as mean ± SD with individual values shown. Statistical significance is denoted as * *p* < 0.05, ** *p* < 0.01, *** *p* < 0.001; ns, *p* ≥ 0.05. Abbreviations: LC, acylcarnitine; HS, heat stress; Tau, taurine. the colors indicate the different LC/Tau treatment combinations: orange, LC−/Tau−; yellow, LC−/Tau+; blue, LC+/Tau−; and purple, LC+/Tau+. In panels C and D, the colors indicate the different LC/HS treatment combinations: orange, LC−/HS−; yellow, LC−/HS+; blue, LC+/HS−; and purple, LC+/HS+.

To further characterize tight-junction protein responses and signaling changes under metabolic stress and heat stress, we measured ZO-1 and occludin protein abundance and assessed the phosphorylation status of p38 and Akt in TM4 Sertoli cells under the indicated treatment combinations (Figure 7). Under acylcarnitine (LC) exposure, ZO-1 abundance was lower than the control condition (*p* < 0.01) (Figure 7A). Taurine treatment alone did not differ from control (*p* > 0.05) (Figure 7A). When taurine was added under LC exposure, ZO-1 abundance was higher than LC alone (*p* < 0.01) (Figure 7A). Occludin showed a similar pattern: occludin abundance was lower under LC than control (*p* < 0.01), taurine alone did not differ from control (*p* > 0.05), and the addition of taurine under LC did not differ from LC alone (*p* > 0.05) (Figure 7B). Representative blots are shown in Figure 7A,B. When LC and heat stress (HS) were evaluated together, ZO-1 abundance was lower under HS than control (*p* < 0.001) and lower under LC than control (*p* < 0.01), with a further reduction when LC and HS were combined (*p* < 0.05) (Figure 7C). Occludin abundance showed the same direction of change: occludin was lower under HS than control (*p* < 0.01), lower under LC than HS (*p* < 0.05), and further reduced under combined LC and HS (*p* < 0.05) (Figure 7D). Representative blots are shown in Figure 7C,D. For signaling readouts, the *p*-p38/p38 ratio was lower under taurine than control (*p* < 0.05), lower under LC than taurine alone (*p* < 0.05), and further lower when taurine was added under LC (*p* < 0.05) (Figure 7E). In contrast, the p-Akt/Akt ratio did not differ between taurine alone and control (*p* > 0.05), was lower under LC than taurine alone (*p* < 0.05), and was higher when taurine was added under LC than under LC alone (*p* < 0.05) (Figure 7F). Representative blots are shown in Figure 7E,F.

## 4. Discussion

HS poses a systemic physiological challenge that disrupts various aspects of reproductive function, notably affecting the seminal plasma microenvironment [33]. Seminal plasma serves as a crucial medium for supporting sperm function and is involved in antioxidant defense, hormone transport, immune modulation, and metabolic support [34]. While previous studies have primarily focused on oxidative stress and changes in specific metabolites in seminal plasma under HS conditions, research on the mechanisms underlying the reconstruction of systemic metabolic networks remains limited. In this study, metabolomic analysis highlighted significant alterations in the expression profiles of lipid metabolites in seminal plasma following taurine intervention under HS conditions, indicating its potential as a systemic metabolic modulator. Our findings substantiate the notion that taurine plays a role in metabolic restructuring by modulating acylcarnitines and other lipid-related metabolites. Acylcarnitines serve as intermediates in fatty acid β-oxidation, with their levels reflecting mitochondrial lipid metabolic efficacy and energy status [35]. Excess accumulation of medium- and long-chain acylcarnitines in seminal plasma can be interpreted as a signature of incomplete mitochondrial fatty acid oxidation (lipid metabolic “traffic jam”); at pathophysiological levels, long-chain acylcarnitines can impair mitochondrial oxidative phosphorylation and promote ROS generation, and their amphiphilic nature may perturb lipid bilayers, thereby increasing the risk of membrane destabilization [36,37,38]. Interestingly, although ROS and MDA were elevated under heat stress, SOD activity also increased, which may reflect a compensatory antioxidant response to enhanced oxidative challenge rather than improved redox homeostasis. In our study, numerous acylcarnitines were significantly elevated in the HS group, while taurine treatment effectively normalized their levels, indicating taurine’s potential to promote lipid metabolic flow and mitigate the risk of lipid accumulation. Moreover, taurine is implicated in glutathione-dependent antioxidant defenses and broader sulfur-related metabolic pathways that support redox homeostasis and metabolic balance [39,40,41]. Previous studies have demonstrated that taurine acts as a precursor for glutathione biosynthesis, augmenting cellular antioxidant capacity [41]. In this study, taurine demonstrated a significant reduction in ROS and MDA levels, along with stabilized SOD activity, likely by activating antioxidant pathways and alleviating lipid peroxidation. Notably, our findings suggest that taurine functions not only as a conventional antioxidant but also as a “metabolic modulator” capable of reconstructing the metabolic landscape under HS conditions and restoring the overall metabolic distribution in seminal plasma. Furthermore, taurine’s effects on endocrine function, including the normalization of testosterone levels, suggest a comprehensive and integrative mode of action. This attribute presents novel opportunities for the nutritional modulation of breeding males facing thermal stress conditions.

Tight junctions between Sertoli cells are integral to blood–testis barrier function and to maintaining the protected testicular microenvironment required for spermatogenesis, yet they are susceptible to disruption by heat exposure [42]. Core TJ proteins such as occludin and ZO-1 are crucial for sustaining this intercellular architecture [11]. Previous research has demonstrated that brief scrotal hyperthermia (e.g., 43 °C for 30 min) disrupts blood–testis barrier tight junctions by reducing occludin/ZO-1 expression and altering their junctional localization, and is accompanied by germ cell loss/apoptosis and impaired spermatogenesis [11,43]. In the present study, taurine treatment significantly restored the expression levels of occludin and ZO-1 in the testes of heat-stressed mice, indicating its potential to preserve junctional integrity under thermal stress conditions.

This protective effect may involve two complementary mechanisms. Firstly, as a physiological osmolyte, taurine can stabilize membrane potential and reduce osmotic stress, potentially preventing the depolymerization and internalization of tight-junction (TJ) proteins induced by heat exposure. Secondly, taurine’s antioxidative properties may attenuate oxidative damage to junctional proteins. Heat stress elevates oxidative and inflammatory stress in the testis (including increased ROS and TNF-α), and is associated with stress kinase activation (e.g., p38 MAPK) and loss/mislocalization of blood–testis barrier tight-junction proteins such as ZO-1 and occludin [12,44,45,46]. Therefore, the antioxidant and anti-inflammatory activities of taurine likely play a crucial role in preserving the structural integrity of Sertoli cell junctions during HS.

In addition to structural protection, taurine (and its precursor hypotaurine) has been implicated in supporting the functional maturation of sperm. Successful fertilization depends not only on adequate motility and normal morphology, but also on capacitation, a coordinated biochemical and biophysical transition that remodels the sperm plasma membrane, activates signaling cascades (including protein kinase-dependent tyrosine phosphorylation), and confers competence for zona pellucida interaction and downstream fertilization events. Consistent with this framework, taurine/hypotaurine can be taken up by sperm via the taurine transporter and has been linked to the maintenance of sperm function under capacitating conditions, supporting fertilization-related endpoints [47,48,49,50]. Tyrosine phosphorylation (p-Tyr) is widely used as a molecular hallmark of sperm capacitation, and higher capacitation-associated p-Tyr is associated with enhanced zona pellucida binding and fertilizing competence [51,52]. In this study, taurine significantly enhanced p-Tyr levels in sperm from heat-stressed mice, enhancing their capacity to bind to the zona pellucida and achieve successful cleavage, indicating improved capacitation and fertilization potential.

Collectively, these findings indicate that taurine mitigates male reproductive impairment induced by HS through dual mechanisms: by stabilizing the testicular microenvironment through the protection of Sertoli cell junctions, and by enhancing sperm functional maturation and fertilization capacity. These synergistic effects underscore the therapeutic promise of taurine as a multi-target intervention for addressing heat-induced male infertility.

Several limitations of this study should be acknowledged. Although dietary taurine supplementation was associated with improvements in semen quality, seminal plasma biochemical indices, and metabolomic profiles in dairy goat bucks under summer field conditions, the number of healthy breeding bucks suitable for long-term repeated semen collection under commercial farm conditions was inherently limited. Accordingly, the goat study was conducted with six animals per group, and the present findings should be interpreted with appropriate caution. Although dietary taurine supplementation was associated with improvements in sperm quality, seminal plasma biochemical indices, and metabolomic profiles in heat-stressed dairy goat bucks, the present study did not include direct in vivo fertility verification, such as artificial insemination outcomes or pregnancy rates. Therefore, the effects of taurine on reproductive performance should be interpreted with caution and regarded as preliminary. The complementary mouse and TM4 cell experiments were included to provide mechanistic support under controlled conditions, but they do not constitute definitive causal evidence in dairy goat bucks. Future studies should further validate these findings using direct fertility endpoints and more targeted mechanistic approaches.

## 5. Conclusions

Taurine supplementation alleviated heat stress-induced declines in sperm quality in dairy goat bucks by modulating seminal plasma metabolism and was associated with improved redox balance and endocrine status. Complementary mouse and Sertoli cell data suggest that taurine may partially restore tight-junction proteins, particularly ZO-1, under long-chain acylcarnitine-related stress, thereby helping to protect the Sertoli cell microenvironment and improve sperm quality. Overall, taurine mitigates heat stress-induced declines in sperm quality in dairy goat bucks by modulating seminal plasma metabolism.

## Data Availability

The data supporting the findings of this study are available within the article. Raw metabolomics data and analysis workflows will be deposited in a public repository and the accession information will be provided in the published article. Until then, data are available from the corresponding author upon reasonable request.

## Supplementary Materials

The following supporting information can be downloaded at: https://www.mdpi.com/article/10.3390/ani16071086/s1, Table S1: Acylcarnitine metabolites identified in seminal plasma of dairy goat bucks.
